# Equity-Centered Postdischarge Support for Medicaid-Insured People: Protocol for a Type 1 Hybrid Effectiveness-Implementation Stepped Wedge Cluster Randomized Controlled Trial

**DOI:** 10.2196/54211

**Published:** 2024-03-26

**Authors:** J Margo Brooks Carthon, Heather Brom, Marsha Grantham-Murrillo, Kathy Sliwinski, Aleigha Mason, Mindi Roeser, Donna Miles, Dianne Garcia, Jovan Bennett, Michael O Harhay, Emilia Flores, Kelvin Amenyedor, Rebecca Clark

**Affiliations:** 1 University of Pennsylvania Philadelphia, PA United States; 2 Penn Medicine at Home Philadelphia, PA United States; 3 Pennsylvania Hospital Philadelphia, PA United States; 4 Penn Center for Community Health Workers Philadelphia, PA United States; 5 University of Pennsylvania Health System Philadelphia, PA United States

**Keywords:** health care disparities, evidence-based practice, Medicaid, transitional care, implementation science, socioeconomic disparities in health

## Abstract

**Background:**

Disparities in posthospitalization outcomes for people with chronic medical conditions and insured by Medicaid are well documented, yet interventions that mitigate them are lacking. Prevailing transitional care interventions narrowly target people aged 65 years and older, with specific disease processes, or limitedly focus on individual-level behavioral change such as self-care or symptom management, thus failing to adequately provide a holistic approach to ensure an optimal posthospital care continuum. This study evaluates the implementation of THRIVE—an evidence-based, equity-focused clinical pathway that supports Medicaid-insured individuals with multiple chronic conditions transitioning from hospital to home by focusing on the social determinants of health and systemic and structural barriers in health care delivery. THRIVE services include coordinating care, standardizing interdisciplinary communication, and addressing unmet clinical and social needs following hospital discharge.

**Objective:**

The study’s objectives are to (1) examine referral patterns, 30-day readmission, and emergency department use for participants who receive THRIVE support services compared to those receiving usual care and (2) evaluate the implementation of the THRIVE clinical pathway, including fidelity, feasibility, appropriateness, and acceptability.

**Methods:**

We will perform a sequential randomized rollout of THRIVE to case managers at the study hospital in 3 steps (4 in the first group, 4 in the second, and 5 in the third), and data collection will occur over 18 months. Inclusion criteria for THRIVE participation include (1) being Medicaid insured, dually enrolled in Medicaid and Medicare, or Medicaid eligible; (2) residing in Philadelphia; (3) having experienced a hospitalization at the study hospital for more than 24 hours with a planned discharge to home; (4) agreeing to home care at partner home care settings; and (5) being aged 18 years or older. Qualitative data will include interviews with clinicians involved in THRIVE, and quantitative data on health service use (ie, 30-day readmission, emergency department use, and primary and specialty care) will be derived from the electronic health record.

**Results:**

This project was funded in January 2023 and approved by the institutional review board on March 10, 2023. Data collection will occur from March 2023 to July 2024. Results are expected to be published in 2025.

**Conclusions:**

The THRIVE clinical pathway aims to reduce disparities and improve postdischarge care transitions for Medicaid-insured patients through a system-level intervention that is acceptable for THRIVE participants, clinicians, and their teams in hospitals and home care settings. By using our equity-focused case management services and leveraging the power of the electronic medical record, THRIVE creates efficiencies by identifying high-need patients, improving communication across acute and community-based sectors, and driving evidence-based care coordination. This study will add important findings about how the infusion of equity-focused principles in the design and evaluation of evidence-based interventions contributes to both implementation and effectiveness outcomes.

**International Registered Report Identifier (IRRID):**

DERR1-10.2196/54211

**Trial Registration:**

ClinicalTrials.gov NCT05714605; https://clinicaltrials.gov/ct2/show/NCT05714605

## Introduction

### Background

Complex care management provides critical support in the days and weeks following hospitalization for individuals with multiple chronic conditions. This postdischarge support can be especially beneficial for nearly 80 million individuals in the United States who are insured by Medicaid [1] and experience higher rates of chronic illness [2], more frequent hospitalizations, and worse clinical outcomes following discharge [3-5]. Readmissions among adults insured by Medicaid ages 45-64 years stand at 24% compared to 20% for older adults insured by Medicare [6]. Similarly, about 34% of individuals insured by Medicaid will experience an emergency department (ED) visit annually, far exceeding the rates of those insured commercially [7]. They experience deficits in primary care assessment and treatment and fewer preventive treatments and guideline-concordant care [8,9]. Thus, Medicaid-insured patients may present to the hospital with poorly managed chronic medical conditions compared with other patients and subsequently require more intensive transitional care coordination in the aftermath of a hospitalization. The need to improve support for Medicaid-insured people following hospitalization prompted our development of the THRIVE clinical pathway. THRIVE is a clinical pathway that supports Medicaid-insured individuals with multiple chronic conditions transitioning from hospital to home by coordinating care, standardizing communication, and addressing unmet needs following hospital discharge, including the social determinants of health.

Most existing postdischarge or transitional care programs were not developed to attend to the specific needs of people insured by Medicaid and instead support older adults or patients with specific chronic illnesses such as heart failure. Other postdischarge support programs require the addition of trained health care providers such as advanced practice nurses or patient navigators to coordinate care [10-12]. At least 1 randomized controlled trial (RCT) evaluating the use of community health workers (CHWs) has demonstrated lower rates of rehospitalization for enrolled low-income individuals with significant social needs [13]. Despite facilitating important links to community-based services, CHWs are unable to manage clinical needs [13-15]. Other postdischarge support programs such as the Camden Coalition Care Model provide transitional care support to people with significant social needs with high rates of health care use, many of whom are insured through Medicaid [16]. An RCT of the Camden Coalition Care Model demonstrated no differences in readmissions at 180 days between treatment and comparison [17]. The care transitions innovation model also provides transitional care support for economically disadvantaged adults, although an RCT found no reductions in 30-day readmissions or ED use [12]. At least 3 additional studies by Liss et al [18], Balaban et al [19], and Jackson et al [20] featured weekly health coaching, self-management education, and medical home follow-up for medically and socially complex patients, although the results of these interventions have been mixed. Inconsistent findings among existing postdischarge support programs suggest the need for alternative innovations that focus on systemic and structural barriers in health care delivery.

Similar to other inequities, posthospitalization disparities among people insured by Medicaid can be traced to a long history of institutional and economic inequities impacting care delivery and outcomes. Racial and ethnically minoritized populations are overrepresented in Medicaid compared to other forms of insurance, with approximately half of Medicaid enrollees younger than 65 years of age being members of racial and ethnically diverse backgrounds [21]. With incomes 138% below the federal poverty level [22], people insured by Medicaid experience the impact of the social determinants of health, including higher levels of financial strain and concerns over out-of-pocket costs, and experience more challenges in accessing specialists and community-based care [23-28]. People insured by Medicaid are also more likely to experience the impact of systemic and institutional racism. Systemic racism refers to the distribution of goods and services in such a way that advantages one group over another or fails to provide adequate resources in the face of need. Systemic factors such as inadequate discharge planning and poor care coordination similarly contribute to unfavorable posthospitalization outcomes [21,27-29]. Interpersonal racism is often expressed through bias and discrimination and is often expressed through microaggressions and stigmatizing language. Distinct from Medicare, Medicaid has its roots in the public welfare system, which has resulted in significant social stigma. Medicaid beneficiaries have described encounters with medical professionals where they were subjected to racial and socioeconomic prejudice [29,30]. These experiences may result in the avoidance of health care settings altogether and more difficulties accessing timely specialty care, all of which paradoxically increase ED use or lead to avoidable hospitalizations due to delayed management of chronic illnesses [31,32].

Given the long-standing disparities experienced by people insured by Medicaid during and following hospitalization, there is a need for transitional care support focused on systemic and structural barriers to care and social determinants of health. The Centers for Disease Control and Prevention [33] defines health equity as “the state where everyone has a fair and just opportunity to attain their highest level of health.” Using an equity lens draws attention to those at greatest risk to how social and economic strata shape access to material resources while also identifying the impact of marginalizing conditions such as racism and health resource constraints that intersect and result in disparate outcomes [34]. Using an equity lens also emphasizes what Peterson et al [35] refer to as the multiple, intersecting spheres of power, defined as the practices, processes, and policies that determine the distribution and access to resources and opportunities needed to be healthy. These spheres of power intersect with individual factors (eg, personal agency) to produce disparate outcomes. From this perspective, addressing postdischarge disparities requires a focus on optimizing service delivery and interdisciplinary collaborations across settings.

### Codeveloping the THRIVE Clinical Pathway

**Figure 1:**
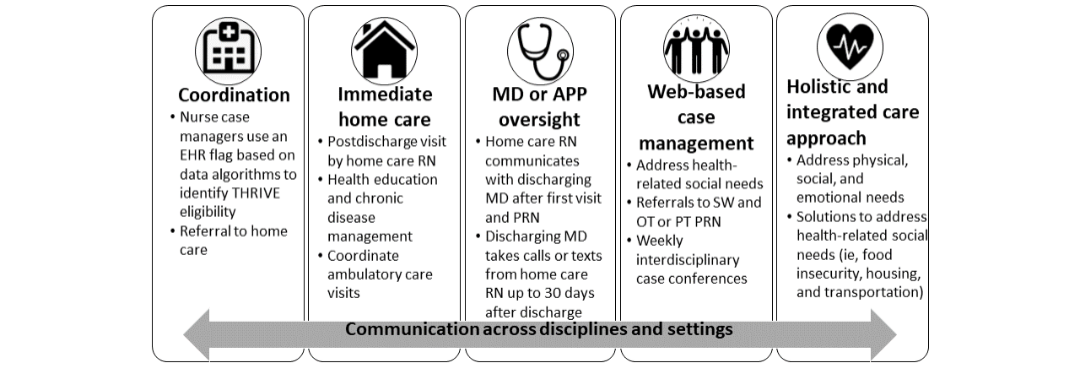
Components of the THRIVE clinical pathway. APP: advanced practice provider; EHR: electronic health record; MD: medical doctor; OT: occupational therapy; PRN: pro re nata (as needed); PT: physical therapy; RN: registered nurse; SW: social worker.

Using an equity lens, our interdisciplinary team of clinicians, researchers, and community members formed to engage in meaningful action to support people insured by Medicaid and transform health care practices and processes that impeded sufficient transitional care support [36]. We began with months of participatory activities informed by an equity lens and principles of human-centered design [37], machine learning [38], and mixed methods approaches [39], which culminated in the development of THRIVE [40]. The THRIVE clinical pathway was launched in a large Level 1 Trauma Center in Philadelphia in 2019 and includes five core components: (1) the identification of individuals insured by Medicaid and coordination of home care referral during discharge planning while hospitalized; (2) provision of immediate home care services where nurses perform medication reconciliation, intensive teaching, and chronic disease management; (3) continued clinical oversight by discharging physicians; and (4) standardized communication between community and acute care providers via web-based case management that (5) prioritizes health-related social needs such as housing or food insecurity faced by Medicaid-insured patients (Figure 1). Our work as a team is anchored by our advisory board, which is comprised of community members, some of whom are Medicaid insured. The THRIVE Community Advisory Board meets quarterly to meaningfully engage past THRIVE participants, caregivers, and community members to ensure that the program goals are rooted in the vision and values of the community we serve.

In many health care settings, posthospitalization disparities among economically disadvantaged people are linked to personal deficits or behaviors. They are often referred to by their volume of health resource use, invariably referred to as “high-cost high needs,” “superusers,” “frequent flyers,” “noncompliant,” or “bounce backs,” and are essentially blamed for poor outcomes. From this perspective, postdischarge disparities are often regarded as immutable social circumstances or moral failures beyond the purview of medicine’s reach instead of a result of the impact of the social determinants of health, structural determinants such as failures in service delivery (eg, fragmented care coordination), and interpersonal racism [41]. In developing THRIVE, we sought to counter long-standing stigmatizing views and instead directed our focus on re-engineering service delivery and intensifying resources to those most in need while maintaining active involvement with affected community members in programmatic design and sustainability efforts.

Published results from a nonrandomized pilot of the first year of the THRIVE pathway revealed that participants experienced increased connections to postacute care services, including social support, and a 50% decrease in rates of 30-day readmissions and ED use compared to patients receiving standard care [42]. With these early findings, we are now poised to evaluate a scalable and sustainable postdischarge management process in a new setting and with a more rigorous evaluation and attention to organizational factors influencing success.

This study will deploy a type 1 hybrid effectiveness-implementation stepped wedge cluster RCT across a single hospital in Philadelphia [43]. The advantage of this trial design is that it offers an efficient and practical way to introduce THRIVE to new sites while reducing the ethical concerns of withholding an intervention that has shown benefit for economically disadvantaged populations. This study will also be guided by the Exploration, Preparation, Implementation, Sustainment framework; Proctor’s outcomes framework; and the Health Equity Implementation Framework (HEIF) [44-46]. The HEIF adapts the Integrated Promoting Action on Research in Implementation in Health Services framework. The HEIF proposes determinants that are believed to predict the successful and equitable implementation of an intervention. The 3 health equity domains included in the HEIF include cultural factors, clinical encounter factors, and societal context [47]. Our use of the framework will help us to detect any factors that might lead to uneven referrals to THRIVE; disparate benefits to the intervention; and any barriers that might prevent organizations, health care providers, or administrators from engaging in the intervention. In their recently published paper, Brownson et al [48] noted that “Incorporating a strong equity focus in implementation science requires not only a deliberate emphasis on the needs, culture, and history of the populations and communities but also more critical analyses and deeper understanding of systems and policies, including care delivery and provider attitudes from which inequities might arise.” They propose action steps for making health equity more prominent in implementation science. To that end, we incorporate and adapt our study design to ensure principles of equity in the approach to data collection, measures, contextual alignment, and dissemination practices (Table 1).

**Table 1:** Equity-relevant domains included in the implementation and outcome evaluation of THRIVE [48].

Domain	Recommendation	Core elements within this study
Evidence base	Link social determinants with health outcomes	Implement THRIVE for all Medicaid-insured individuals at a new study site to meet the postdischarge clinical and social needs of an economically disadvantaged population.
Methods and measures	Integrate equity into implementation models	Incorporate the 3 domains of the Health Equity Implementation Framework into the implementation evaluation.
Context	Engage organizations in internal and external equity efforts	Engage with clinical partners, administrators, and community advisory board members to implement THRIVE at a new site.
Cross-cutting issues	Focus on equity in dissemination efforts	Plan activities with THRIVE Community Advisory Board to disseminate study results.

### Aims and Hypotheses

The specific aims of this research are to:

1. Examine referral patterns, 30-day readmission, and ED use patterns for participants who receive THRIVE support services compared to those receiving usual care.
  - Hypothesis 1: We anticipate that referral patterns will increase and that 30-day readmissions and ED use will decrease for THRIVE participants compared to usual care.
2. Evaluate the implementation of the THRIVE clinical pathway, including fidelity, feasibility, appropriateness, and acceptability.

## Methods

### Overview

Our study methods are described per the SPIRIT (Standard Protocol Items: Recommendations for Interventional Trials) statement and the CONSORT (Consolidated Standards of Reporting Trials) extension for stepped wedge cluster RCTs [49,50]. The SPIRIT and CONSORT checklists are included as Multimedia Appendices 1 and 2.

### Study Setting

The study setting includes a single site—Pennsylvania Hospital (PAH), the nation’s first acute care setting in the country and 1 of 6 hospitals in the University of Pennsylvania Health System. More than 50% of PAH’s patient population on the hospitalist service self-identify as racial or ethnic minority individuals, and >25% are Medicaid insured [34]. PAH provides over 40,000 ED visits and >18,000 adult admissions annually.

### Design and Randomization

#### Overview

This is a prospective single-site type 1 hybrid effectiveness-implementation pragmatic stepped wedge cluster RCT. Our stepped wedge design will include a sequential randomized rollout of the intervention to case managers at PAH in 3 steps, and data collection will occur over 18 months. This mixed methods (quantitative and qualitative) study design involves simultaneous collection and analysis of quantitative and qualitative data, giving priority weight to the quantitative data to evaluate program referrals, outcomes, and program fidelity, while qualitative data will evaluate the process through detailed descriptions of perspectives of barriers and facilitators faced by health care providers in implementing the THRIVE intervention [51]. This study includes qualitative interviews and surveys of clinicians and administrators to assess their perspectives on appropriateness, feasibility, and acceptability of THRIVE. Nesting the qualitative interviews within an RCT of the THRIVE intervention will allow us to determine whether the intervention improved primary outcomes (referrals to home care, 30-day readmission, ED use, and connection to primary care providers) and to identify professional and organizational barriers to implementation. Combining these insights with effectiveness outcome data will allow consideration for meaningful contextual factors that are considered critical to the implementation of THRIVE and subsequent outcomes.

#### Randomization

Groups of case managers (4 in the first group, 4 in the second, and 5 in the third) were randomly assigned by the study methodologist to each sequence using a random number generator. A stepped wedge design—that is, a 1-time crossover design—was used for THRIVE (Table 2).

Specifically, following a baseline data collection period, 4 case managers will be randomized to receive training on the THRIVE clinical pathway. Following training, they begin referring to the THRIVE clinical pathway. At 8-week intervals, the remaining case managers will be trained in the THRIVE intervention and will be able to begin submitting referrals. Since case managers from the sequences work within the same space, the ability to conceal allocation was limited; however, all case managers were made aware of their upcoming ability to refer to the THRIVE clinical pathway.

**Table 2:** Study design for the type 1 hybrid effectiveness-implementation stepped wedge cluster randomized controlled trial of the THRIVE clinical pathway.

Cluster	Baseline (3 months)	Enrollment period 1 (8 weeks)	Enrollment period 2 (8 weeks)	Enrollment period 3 (8 weeks)	Follow-up (18 months)
Sequence 1 (4 case managers)		✓	✓	✓	✓
Sequence 2 (4 case managers)			✓	✓	✓
Sequence 3 (5 case managers)				✓	✓

### THRIVE Eligibility Criteria

Individuals eligible for a THRIVE referral include (1) being Medicaid insured, dually enrolled in Medicaid and Medicare, or Medicaid eligible; (2) residing in Philadelphia; (3) having experienced a hospitalization at the study hospital for more than 24 hours with a planned discharge to home; (4) agreeing to home care at partner home care settings; and (5) being aged 18 years or older. If home care services are declined at any time following discharge, THRIVE services are also discontinued. If palliative or hospice services are ordered following discharge, THRIVE services will not be offered.

### Clinician Eligibility and Recruitment

Nurse case managers (CMs), home care nurses, physicians, advanced practice providers, and administrators who are actively involved in supervisory roles or in referring to THRIVE will be invited to provide feedback on the appropriateness, accessibility, feasibility, and workload involved in referring to the THRIVE clinical pathway. During the consenting process, the research coordinator will obtain preferences for the best communication method to schedule the interview (eg, email or telephone call). A total of 22 interviews and surveys are planned. Participants who complete both the surveys and interviews will receive a US $90 gift card by mail.

### Ethical Considerations

This study was approved by the University of Pennsylvania’s Institutional Review Board (IRB #4 approved this protocol #852910 on March 10, 2023). The study will be conducted according to the Declaration of Helsinki and national regulations. Study participation is voluntary, and written consent will be obtained prior to clinician interviews. Our IRB approval includes a HIPAA (Health Insurance Portability and Accountability Act) waiver of informed consent for THRIVE participants’ clinical data. All study data collected will be deidentified, pseudonyms will be used, and data will only be accessible to the research team and stored on password-protected computers. Clinicians will be compensated US $90 by gift card for interview participation. Individual participants will not be identifiable in any published material.

### Data Collection and Management

#### THRIVE Participants and Usual Care Group

Characteristics of THRIVE participants captured in weekly case conferences (eg, home care services, social determinants of health identified and met, and community-based services) will be stored on a Research Electronic Data Capture (REDCap; developed by Vanderbilt University) database. Data will be derived from the patient electronic health record (EHR) and hospital billing system to compare the postdischarge outcomes of participants receiving THRIVE compared to those who do not. These data will be deidentified, and a HIPAA waiver of informed consent was obtained by the University of Pennsylvania’s IRB. Data will be stored on a secure password-protected data server, and access will be provided to the study statisticians (MOH), who will be blinded to the assignment of the intervention. All aspects of study design, database integrity, and study conduct will be overseen by our data and evaluation core team, which includes doctorly prepared health service researchers and statisticians.

#### Clinicians and Administrators

**Figure 2:**
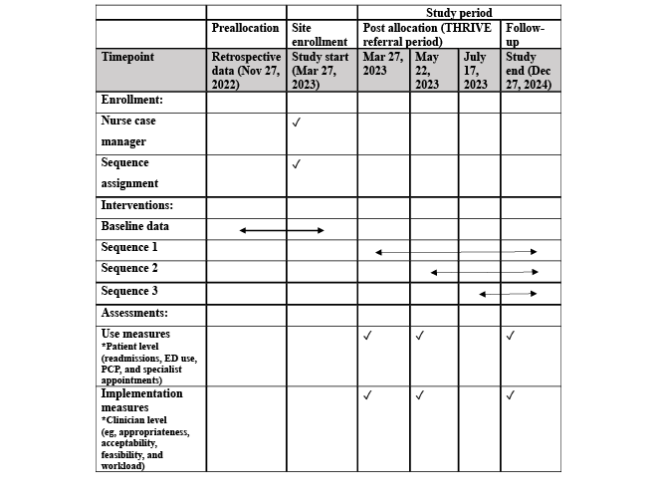
SPIRIT (Standard Protocol Items: Recommendations for Interventional Trials) diagram—THRIVE schedule of evaluations. ED: emergency department; PCP: primary care provider.

Case managers at PAH will be recruited and enrolled in the study per the stepped wedge design. At 8 weeks and 18 months, we will survey clinicians via Qualtrics (Qualtrics) and convene one-on-one interviews (or focus groups depending on clinician availability). Interviews will be conducted in the setting of the clinician’s choice, either in a conference room in the hospital or via Zoom (Zoom Video Communications). During interviews, clinicians and administrators will be asked for permission to audio record the interview. If the participant agrees, all conversations from that point forward will be recorded. If the clinician or administrator consents to the interview but does not agree to recording, to retain the participant, the interviewer will take detailed notes during the conversation instead of recording. Interview documents are labeled with a unique identification number stored separately for identifying information about the participant. The study timeline is outlined in a SPIRIT diagram (Figure 2).

### Sample Size

We anticipate a sample size of 534 inclusive of THRIVE referrals and the comparison group (derived from the EHR). Based on a power analysis, a sample size of 472 (236 THRIVE participants and 236 comparisons) for the outcome of readmissions, or a sample of 534 (267 THRIVE participants and 267 comparisons) for the outcome of ED visits, will yield 80% power to detect significance at the .05 level. We will target a sample size of 534 to ensure adequate power for both outcomes. We anticipate consenting 22 clinicians and administrators to complete surveys and interviews to provide data for implementation outcomes.

### The THRIVE Intervention

#### Current Usual Care

To provide evidence of the effectiveness and implementation of THRIVE, we will compare individuals receiving THRIVE services to patients receiving usual care. The current usual care will serve as the control condition that patients will receive during the control phases of the design. Usual care may include discharge care planning that reflects the patient’s medical needs, which may or may not include referrals to home care, skilled nursing or rehabilitation facilities, and other outpatient services.

#### Enrollment in THRIVE

Enrollment in THRIVE begins with identification by CMs during hospitalization using a validated predictive algorithm developed by our team [31]. The algorithm is housed in the EHR and leverages principles of behavioral economics to “nudge” CMs to initiate a THRIVE referral. The THRIVE EHR flag was introduced to alleviate the need for CMs to manually identify THRIVE participants.

Once identified, THRIVE participants will receive a home care referral to Penn Medicine at home care services. The initial home care visit will be scheduled and completed by a nurse within 48 hours of discharge, at which time, medications are reconciled, discharge orders are reviewed, and person-centered goals of care are set. During the initial evaluation, home care nurses will determine the frequency of visits deemed necessary, with weekly visits ranging from 1 to 3 times depending on needs. Participants may receive additional services, such as speech or occupational therapy as required. All THRIVE participants receive a social work referral, their psychosocial needs are further assessed, and connections to community resources are provided. THRIVE participants receive a CHW referral as deemed necessary.

As a part of the THRIVE pathway, physician colleagues will extend continuity of care into the home following discharge to all THRIVE participants. Home care nurses will provide a brief patient status update to discharging physicians following the first home visit. During calls, nurses can raise questions about medications, clarify discharge orders, and receive guidance on emerging symptoms with the provider most recently involved with care. Extended supervision by discharging physicians will be provided for up to 30 days or until THRIVE participants have seen a primary care provider or specialist.

THRIVE participants will receive further support during discussions in our weekly interdisciplinary case management conferences for 4 weeks following discharge. During case management meetings, we will support connections to postacute primary, specialty care, behavioral health, and substance use services as needed. If a source of primary care is absent, we work to link THRIVE participants to community providers. Our intensive support addresses many of the social determinants of health that are known to influence health inequities such as housing, food insecurity, and limitations in access to behavioral health (Figure 1).

#### Implementation Strategies

We will use a series of implementation strategies to improve the effectiveness of THRIVE and promote its use [52]. These strategies will include (1) building and sustaining a coalition of diverse stakeholders through regular meetings and providing updates on THRIVE outcomes; (2) developing educational materials and conducting ongoing training of CMs, physicians, and advanced practice providers in the hospital and nurses and managers in home care using a scripted in-person training module to teach them about the elements of THRIVE and their role in implementing it; (3) auditing and providing feedback through the use of a referral dashboard to monitor all eligible THRIVE participants to document the individuals who were eligible for the intervention and not referred and providing feedback to case managers and document reasons for why referrals did not take place; (4) emailing reminders to case managers at weeks 2, 4, and 6; (5) identifying and preparing champions to promote the use of THRIVE; and (6) engaging of our advisory board to seek feedback for improvements.

### Evaluation

#### Aim 1

Aim 1 is to examine referral patterns, 30-day readmission, and 30-day ED use patterns for participants who receive THRIVE support services compared to those receiving usual care.

##### Outcomes and Measures

The primary outcome includes the referral rate to home care made by CMs compared to baseline and the nonrandomized cluster. The secondary outcomes are 30-day readmissions and ED visits. Additional covariate data at the patient (eg, demographics and comorbidities) and unit levels (eg, type of unit and discharging team or provider) will be collected from analyses. For THRIVE participants, these outcomes will be assessed from the date of hospitalization, which resulted in a referral to THRIVE. For the usual care group, we will identify all readmissions and ED visits occurring after the first index hospitalization identified in the EHR for the calendar year of the study. Outcome measures, guiding framework, data sources, collection, and timing are described in Table 3.

**Table 3:** Summary of outcomes, measures, data sources, collection method, and timing.

Construct and framework					Design		Method of collection			Timing	
**Aim 1: Examine referral patterns, 30-day readmission, and ED^a^ use patterns for participants who receive THRIVE support services compared to those receiving usual care**											
	THRIVE referrals^b^ 30-day readmissions^c^ 30-day ED visits^c^ Primary care visit within 30 days of discharge^c^ Specialty care visit within 30 days of discharge^c^			Stepped wedge cluster randomized controlled trial		Electronic health record			Baseline and at the study end		
**Aim 2: Evaluate the implementation of the THRIVE clinical pathway, including fidelity, feasibility, appropriateness, and acceptability**											
	**Health Equity Implementation Framework (HEIF)**										
		Culturally relevant factors^d^ Clinical encounter^e^ Societal context^f^	Mixed methods approach to determine if HEIF factors are associated with uneven or disparate benefits to the intervention and to assess the implementation of THRIVE at a new site					Interviews with clinicians and administrators involved with THRIVE			8-week postintervention from the start of each sequence and at the study end
	**Exploration, Preparation, Implementation, Sustainment**										
		Adoption^g^ Active implementation^h^	Mixed methods approach to determine the adoption and implementation of THRIVE in a new site					Interviews with clinicians and administrators			8-week postintervention from the start of each sequence and at the study end
	**Proctor implementation outcomes**										
		Acceptability^i^ Appropriateness^j^ Feasibility^k^	Mixed methods approach to determine the Proctor implementation outcomes					Survey and interviews with clinicians and administrators			8-week postintervention from the start of each sequence and at the study end
		Fidelity^l^	Retrospective chart review					Standardized THRIVE checklist			Monthly

^a^ED: emergency department.

^b^Proportion of THRIVE referrals of those who are THRIVE eligible.

^c^Proportion of THRIVE participants experiencing the outcome within 30 days of discharge compared to usual care group.

^d^Organizational commitment to addressing disparities.

^e^Relative advantage of THRIVE to patients, degree of fit with existing practice, competing demands, and bias.

^f^Structures outside of the hospital that affect patient care.

^g^Organizational values, culture embedding, and championing adaption.

^h^Organizational priorities and goals and readiness for change and culture or climate.

^i^How fair or reasonable THRIVE is deemed.

^j^To what extent THRIVE seems suitable.

^k^The practicality and ease of delivering THRIVE.

^l^Fidelity to THRIVE’s core components.

##### Analysis

We will develop monthly reports to monitor the referral patterns to the THRIVE clinical pathway. The target estimate and effect measure is the time-adjusted difference in participant outcomes [53,54]. The analysis of use outcomes (eg, 30-day readmissions and ED use) will use a mixed effects negative binomial regression model with random effects for the medical team and fixed effects for time to account for the stepped wedge cluster randomized design. Sensitivity analyses may be performed using logistic regression for health service use based on the distribution of the data. We will also examine the stability of effect estimates using generalized estimation equations with small sample adjustment, given the study design [55].

##### Equity Evaluation

Our analyses will consider several equity metrics and include interaction terms to capture racial and ethnic differences and biological sex for THRIVE participants compared to patients receiving usual care. Using all payor data obtained from the EHR, we will also conduct a secondary analysis to examine health service use across all insurance payor types for THRIVE participants and the usual care group, allowing a proxy measurement for differences across socioeconomic strata.

#### Aim 2

Aim 2 is to evaluate the implementation of the THRIVE clinical pathway, including fidelity, feasibility, appropriateness, and acceptability.

##### Outcomes and Measures

Fidelity will be assessed using a standardized checklist and monthly report. Surveys and interviews of clinicians and administrators involved with THRIVE will be used to assess the secondary implementation outcomes of interest. These outcomes will be measured twice (at 8 weeks from the start of each sequence and at the end of the study). The surveys will use validated instruments that measure (1) feasibility, practicality, and ease of delivering THRIVE; (2) acceptability, how fair or reasonable THRIVE is deemed; (3) appropriateness, to what extent THRIVE seems suitable; and (4) clinician workload, an objective assessment of the demand of the THRIVE clinical pathway on clinician time. Clinician interviews will assess the determinants of THRIVE implementation using the HEIF [44,45]. Demographics of clinicians and administrators will also be collected.

##### Analysis

Summary statistics will be produced from the quantitative measures of feasibility, acceptability, appropriateness, and clinician workload. All interview data will be analyzed using constant comparative analysis to examine data across cases. Initially, we will use an iterative process of close readings and discussion of a random sample of interview transcripts and will analyze patterns in the recurrence and distribution of emergent concepts across participants. The principal investigator and research coordinator staff will then create a data dictionary and apply codes for themes that emerge while reading what the respondents say. The HEIF will inform the development of the codebook, including definitions and subsequent integration of the qualitative and quantitative data [47]. The integration of the qualitative data will help to extend and expand the findings of the quantitative findings. We will use NVivo (version 12; Lumivero) to manage our data.

### Dissemination

Our team will lead several efforts to promote the dissemination and translation of our findings. First, results will be shared widely through publications, conference presentations, policy briefs, and well-established channels via the Penn School of Nursing Communication Office and the Center for Health Outcomes & Policy Research’s national and international networks. Examples of print and public engagements will include up to 5 publications (at minimum 1-2 papers per aim), annual scientific presentations or conferences, infographics, and guest features on podcasts (eg, AmplifyNursing). We have built a customized THRIVE website with plans to share open-access modules on the components of THRIVE and the process for implementation. We will engage with our THRIVE Community Advisory Board to develop equity-focused dissemination efforts that are system and community focused. For example, we have previously participated in health fairs at local churches. We anticipate that the focus of this project will be of equal interest to other faith-based and community organizations and look forward to collaborating throughout the study period, not just at the end.

## Results

This project was funded in January 2023. We received IRB approval on March 10, 2023. Data collection will occur from March 2023 to July 2024. Results are expected to be published in 2025.

## Discussion

### Expected Findings

We anticipate that patients who participate in the THRIVE clinical pathway will experience fewer 30-day readmissions and ED visits as well as more connections to primary and specialty care compared to usual care. We also expect that clinicians will value and appreciate the additional supports provided to patients with an increased burden of health-related social needs.

### Comparisons With Prior Work

Most current transitional care programs were not developed to meet the needs of people insured by Medicaid and require additional personnel to implement them [10-12]. Several clinical trials of transitional care programs have resulted in mixed findings, with some noting decreased rehospitalization, while others have found no reductions in 30-day readmissions or ED use [12-17]. Similarly, other transitional care models have incorporated interventions including weekly health coaching and self-management education, though findings are inconsistent [18-20].

### Strengths and Limitations

The main strength of this study is the use of a stepped wedge approach. In this design, after all of the case managers have “stepped in” to refer to THRIVE, all eligible patients will be able to receive THRIVE services. A limitation of this study is that it is a single-institution trial. However, we believe this study will yield important findings, given the rigor of the design and the focus on embedding equity-based principles throughout our implementation.

### Conclusions

The goal of THRIVE is to reduce disparities and improve postdischarge care transitions for Medicaid-insured patients through a feasible system-level intervention that is satisfying for THRIVE participants, clinicians, and their teams in hospitals and home care settings. By using our equity-focused case management services and leveraging the power of the electronic medical record, THRIVE creates efficiencies by identifying high-need patients, improving communication across acute and community-based sectors, and driving evidence-based care coordination. This study will advance the field of equity-focused evidence-based interventions by testing both the effectiveness of patients’ health improvement and the adoption of THRIVE by typical clinical practices. It will add important findings about how the infusion of equity-focused principles in the design and evaluation contributes to both implementation and effectiveness outcomes.

## Appendix

Multimedia Appendix 1 SPIRIT (Standard Protocol Items: Recommendations for Interventional Trials) reporting checklist for protocol of a clinical trial.

Multimedia Appendix 2 CONSORT (Consolidated Standards of Reporting Trials) checklist for stepped wedge trials.

Multimedia Appendix 3 Peer Review Feedback from funder.

## Abbreviations

CHW: community health worker
CM: nurse case manager
CONSORT: Consolidated Standards of Reporting Trials
ED: emergency department
EHR: electronic health record
HEIF: Health Equity Implementation Framework
HIPAA: Health Insurance Portability and Accountability Act
IRB: institutional review board
PAH: Pennsylvania Hospital
RCT: randomized controlled trial
REDCap: Research Electronic Data Capture
SPIRIT: Standard Protocol Items: Recommendations for Interventional Trials

## Data Availability

The data that will support the findings of this study is available from the Penn Data Store and the electronic medical record and is not publicly available.
