# Supplementary material for: Equity-Centered Postdischarge Support for Medicaid-Insured People: Protocol for a Type 1 Hybrid Effectiveness-Implementation Stepped Wedge Cluster Randomized Controlled Trial
Source: JMIR Res Protoc. 2024 Mar 26;13:e54211. doi: 10.2196/54211 (PMC11005441; doi:10.2196/54211)
Supplement: Multimedia Appendix 3 [file resprot_v13i1e54211_app3.pdf]

The Rita Hillman and Alex Foundation

200 Park Avenue So., Ste. 1705
New York, NY 10003
Tel. 212-265-3115 | Fax 212-765-3134

CONFIDENTIAL | 2022 CATALYST AWARD

Peer Review Feedback:

Expanding and Evaluating the THRIVE Transitional Care Support Program for Economically Disadvantaged Individuals

J. Margo Brooks Carthon, PhD, RN, FAAN, University of Pennsylvania School of Nursing

---

Review Process

Culled from an initial group of 63 applicants, seven finalists submitted full proposals for evaluation by a review committee from May to September 2022. The review committee, consisting of three nurse scientists from U.S. academic institutions and two Foundation staff members, assessed proposals utilizing the grant program's evaluation framework. The evaluation framework measures the quality and potential impact of proposed projects based on the following criteria: *health impact on marginalized populations*, *innovation and creativity, nursing-driven*, *scalability*, *evidence/execution plan*, *health equity*, *inclusion, institutional/community support*, *leadership*, and *sustainability*.

Overall Assessment

The proposal has a high leverage focus: features one of the nation's preeminent nursing institutions and an accompanying dynamic 'all-star team' when it comes to improving health delivery and addressing health inequities. The team has demonstrated promise with this approach in an earlier iteration and is looking to expand into another inpatient facility. The strategy to join approaches that address health inequities side-by-side with reducing "big-ticket" utilization items such as hospitalization and emergency department use is compelling. The project has high potential to generate new evidence that could help to establish the intervention as a standard for delivering comprehensive transitional care to Medicaid patients and their families. The proposed project is recommended for funding.

The following questions, which emerged during the review of the application, were addressed during a two-hour site visit:

- How is the community and target population involved in the design, implementation, and evaluation of the proposed intervention?
- How will the voice of the patient participants be evaluated and integrated for impact on this

nurse-led intervention for their transitional care in the community?

- What is the composition of the community advisory board and what expertise do they bring?
- Please describe in greater detail the sustainability plan? Specifically, what leadership, systems-level, and policy changes are needed to make the intervention successful long term?
- How will health equity outcomes be measured and evaluated for impact? How will the project demonstrate more direct improvements in health equity for the target population?
- Please describe the unique elements of this intervention. Share more on how it overlaps with or is distinguished from other models that provide transitional care?
- Please discuss the role of advanced care clinicians in the proposed intervention? How does the intervention propose to mitigate the cost-prohibitive barrier to utilizing specialized providers?
- Please describe in greater detail the scalability plan. To what degree have hospital systems on a state/national level shown an interest in the proposed intervention?
- The project is strongly anchored to the hospital setting. Might there also be opportunities to partner with Managed Medicaid Providers or with Federally Qualified Health Centers? Has the project team considered meeting with leaders of these plans to try to engage/collaborate 'upstream' and avoid becoming adversarial?
- Is the leadership team developing a business model for further dissemination?
- Please provide role clarification for the specific health care providers on the team. How can it be ensured that each team member is providing the best care according to their unique skill sets and training (eg. use of a nurse vs a social worker)?
- Please describe how this intervention is innovative? Does innovation rest on the identification of and service provided to a target population that is often overlooked? What is intrinsically innovative about the model or the structure of the intervention that can be replicated?
